# Police Officers’ Interrogation Expertise and Major Objectives in Police Service and Training: A Comprehensive Overview of the Literature

**DOI:** 10.3389/fpsyg.2022.823179

**Published:** 2022-06-01

**Authors:** Markus M. Thielgen, Stefan Schade, Patrick Niegisch

**Affiliations:** ^1^Area of Study VIII – Social Sciences, Department I – University Education, Rhineland-Palatinate Police University, Büchenbeuren, Germany; ^2^Criminal Investigation Department of Wittlich, Police Headquarter of Trier, Rhineland-Palatinate State Police, Wittlich, Germany

**Keywords:** interrogation, interviewing, job and training performance, competence, interrogation and interview concepts, communication and interaction skills, police training and expertise

## Abstract

Interrogation is a core task of practical police work. The outcomes of interrogation often provide crucial evidence for solving criminal cases. The success of interrogation depends on interactions between police officers and citizens. Based on a comprehensive literature overview, we propose a three-factor typology for interrogations by police officers. First, the *competencies* of police officers refer to the application of personal, professional, social, and methodological capabilities. The underlying *concept* of interrogation refers to the application of both explicit and implicit experience-based interrogation models. *Communication* refers to the goal-directed application of communication tactics and techniques. According to this typology, we discuss the major objectives of police interrogation in police service and training from police officers’ perspectives. The present study provides guidance for practical police services and training by offering an evidence-based interrogation standard.

## Introduction

Interrogation is a core task of practical police work that is conducted almost daily. It is indispensable for solving criminal cases; they may even deliver a single piece of key evidence for a criminal case and determine the outcome of criminal proceedings. Therefore, determining the factors that contribute to a successful interrogation is of value. Since 2010, there has been an ongoing debate about what constitutes a *good* interrogation, in both research and practice in Germany (e.g., Sticher, 2007; Holzhauer, 2016) and internationally (e.g., Snook et al., 2021; cf. Laney and Loftus, 2016). In this study, we aimed to focus on the evidence-based factors that underlie interrogation success in police services.

Assuming that interrogation success results from the interaction between police officers and citizens, we propose a three-factor typology of interrogation expertise of police officers based on a comprehensive literature overview. Within this typology, the *competencies* of police officers refer to the application of personal, professional, social, and systematic capabilities. The underlying *concepts* of interrogation refer to the application of both explicit and implicit experience-based interrogation models. *Communication* refers to the goal-directed application of interrogation tactics and techniques. Based on the competencies, concepts, and communication, social dynamics with citizens may determine the success or failure of interrogations, with a specific focus on the knowledge, skills, abilities, and experience of the interrogator, their interactions with civilians, and the management of contextual factors. In line with this typology, we discuss the major objectives of police interrogation in police service and training from the perspective of police officers. Thus, the present study provides guidance on practical police services and training by offering an evidence-based interrogation standard. Furthermore, we encourage debate within police organizations regarding evidence-based interrogation standards in police services and training and discuss implications for personnel selection and police training.

In the present paper, we provide a definition of the terms “interrogation” and “interrogation success” as a result of the interaction between the police officer and citizen. We may focus on police officers’ interrogation expertise and illustrate the factors that contribute to favorable interrogation outcomes. Specifically, we focus on the strategies and methods that promote interrogation success based on recent comprehensive literature on police interrogation in Europe and worldwide.

**FIGURE 1:**
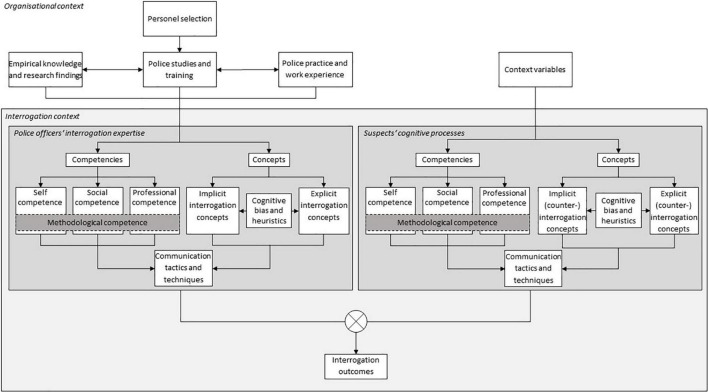
Interrogator-interrogee-interaction model.

We propose a three-factor typology of police officers’ interrogation expertise: competencies, concepts, and communication. Contextual factors may significantly affect the development of police officers’ interrogation expertise. The competencies, concepts, and communication tactics and techniques within the interrogation interaction process are presented in Figure 1. These factors refer to the decision-making process of identifying and applying communication tactics and techniques based on individual competencies and (implicit or explicit) interrogation concepts to meet the demands of the complex and dynamic context of interrogation.

## Interview and Interrogation

In contrast to police *interviews*, scholars may characterize *interrogations* according to goal orientation, the purpose of criminal prosecution, a criminal prosecution institution carrying out the interrogation, and the official function of the police officer that is transparent to the citizen (i.e., the formal definition of interrogation; Artkämper and Schilling, 2018; Märkert, 2021a,b; Niegisch and Thielgen, 2022). From a criminalistic perspective, interrogations are the methods that can be applied by police officers to gather valid information about the criminal act, the crime scene, both the alleged perpetrators and victims, and the context that may help to derive alternative hypotheses of the genesis of the criminal act (i.e., the criminalistic definition of interrogation; Artkämper and Schilling, 2018; Märkert, 2021a,b; Niegisch and Thielgen, 2022). Generally, the interrogation literature differentiates between collaborative and information-gathering approaches (e.g., the PEACE-model; Milne et al., 2003). Interrogations may be conceptualized as either a method of information acquisition (i.e., a collaborative approach) or a competitive interaction to obtain a confession (i.e., an accusational approach, such as the REID-method; Inbau et al., 2013). Consequently, interrogations can yield several outcomes. Of all the relevant information that can be acquired for criminal cases, confessions represent one possible outcome of an interrogation. Police officers may adopt either a more collaborative or more confrontative interrogation strategy (Niegisch and Thielgen, 2022).

## Interrogation Success

*Interrogation success* can be defined from two perspectives. From a work and organizational point of view, interrogations may be considered a complex task that requires a continuous adjustment of one’s actions to achieve a goal (cf. Frese and Zapf, 1994). Firstly, interrogation success may reflect a fit between work-related outcomes and expectations of both police organizations and significant actors, such as citizens, prosecutors, and judges (so-called “in-role” behavior; Campbell et al., 1993). Interrogation success may also be derived from the personal initiative or the prosocial behavior of the interrogator (so-called “extra-role” or citizenship behavior; Organ and Ryan, 1995). Finally, interrogation success may be characterized as the absence of counterproductive behaviors, such as legal violations (Dilchert et al., 2007; Marcus et al., 2016) or even torture (Rantamäki et al., 2020).

From a psychological diagnostics perspective, interrogation refers to a diagnostic method that provides questionnaire data [“Q-data,” in contrast to life record or observation data (“L-data”) or test data (“T-data”)] (Cattell, 1957). Consequently, quality criteria of psychological diagnostics may be applied (Davidshofer and Murphy, 2005; Moosbrugger and Kelava, 2012; Schmidt-Atzert and Amelang, 2012). Concerning objectivity and reliability, the outcome of interrogation must be free of individual influence from the interrogating police officer. Furthermore, interrogation outcomes must be error-free because legal errors can lead to the prohibition of the evidence in court. Tactical and communication errors may result in lower quantity and quality of information obtained, and erroneous statements or even false confessions. Adverse communication styles may reduce the trust and motivation to collaborate with the interrogator, which can lead to refusal to provide evidence. In terms of content validity, an interrogator’s questions must relate to the criminal case, i.e., interrogations must offer case-solving information. Finally, given the limited time and personnel resources, the criterion of efficiency is relevant. In minor cases, effective yet pragmatic interrogation practices may be appropriate. However, in most cases, the criterion of effectivity must be prioritized over the criterion of efficiency when defining an interrogation strategy (Dando et al., 2008).

Recently, research has become increasingly focused on interrogation outcomes. For instance, Cabell et al. (2020) proposed an “effect taxonomy” of interrogation techniques by defining four categories of how different interrogation techniques influence guilty and innocent suspects: confession-prone (an interrogation technique that increases the likelihood of confessing for both guilty and innocent suspects), differentiating (a technique that increases the likelihood of confessing for guilty suspects but not for innocent suspects), assimilating techniques (a technique that increases the likelihood of confessing for innocent suspects but not for guilty suspects), and nonconfession-prone (a technique that does not increase the likelihood of confessing for either guilty or innocent suspects). Moreover, a recent study investigated jail inmates’ attitudes toward police officers’ interrogation practices. Specifically, suspects endorsed interrogation strategies characterized by respect, dignity, voice, and commitment to the truth. In contrast, they reported aversion toward the false evidence ploy and approaches that involve aggression (Cleary and Bull, 2018). Therefore, we propose:

*Proposition 1a. Interrogation success can be described by aspects of in-role and extra-role behavior, the absence of counterproductive behavior, and favorable attitudes towards interrogation strategies.*

*Proposition 1b. Interrogation success meets the criteria of objectivity, reliability, validity, and efficiency.*

## Police Officers’ Interrogation Expertise

Based on a comprehensive literature review, we propose a three-factor typology of police officers’ interrogation expertise: competencies, concepts, and communication. First, the competencies of police officers refer to the application of personal, professional, social, and systematic capabilities. Specifically, *competencies* represent the general, broadly defined, and relatively stable capacities of an individual, who may excel in a wide range of police work tasks, including interrogation. Although competencies are considered non-specific with respect to interrogations, recent models of interrogation need to be extended to include competencies (Kelly et al., 2013, 2015, 2016; Meissner et al., 2021). Indeed, individual attributes, such as cognitive abilities (e.g., working memory capacity) and personality traits (e.g., emotional stability), are largely untrainable; therefore, they are a matter of personnel selection of interrogators (Schuler, 2000; Thielgen, 2016; Nettelnstroth et al., 2019). Moreover, competencies enable police officers to apply both interrogation concepts (e.g., extended cognitive interviews) and communication tactics (e.g., questioning techniques) more effectively (Paulo et al., 2013).

The underlying *concepts* of interrogation refer to applying both explicit and implicit experience-based interrogation models. Specifically, concepts refer to either conscious or unconscious action plans that guide police officers throughout an interrogation. The consideration of concepts may also extend to recent research on police interviewing and interrogation (e.g., Kelly et al., 2013, 2015, 2016; Meissner et al., 2021) because they represent a strategic meta-perspective that determines the use of tactics and techniques. In contrast to the relative stability of competencies, it may be easier to address concepts via interrogation training. In particular, concepts could provide inexperienced police officers with structured guidance around conducting interrogations successfully. During the course of an interrogation, explicit concepts may help police officers control communication both strategically and tactically (Paulo et al., 2013).

*Communication* refers to the goal-directed application of communication tactics and techniques. The use of a wide range of nonverbal and verbal communication techniques promotes the ability, motivation, and performance of testimony and confessions in the context of police interrogations. Communication tactics and techniques have also been proposed by recent taxonomies of police interviews and interrogations (e.g., Kelly et al., 2013, 2015, 2016). Similar to concepts, communication practices are subject to interview and interrogation training support for police officers to achieve interrogation success (Meissner et al., 2021). An interrogator’s conceptual approach determines the strategic and tactical communication approach, whereas communication techniques themselves are a concrete operational application of communication plans (e.g., Kelly et al., 2013, 2015, 2016; Meissner et al., 2021).

The social dynamics of police-citizen encounters may determine the success or failure of interrogations depending on the competencies, concepts, and communication. We propose that (1) a successful interrogator possesses a variety of distinct competencies, (2) the interrogator establishes the interrogation based on an elaborate concept, and (3) the interrogator applies a bandwidth of well-trained communication techniques. Thus, competencies may represent the macro-perspective of interrogation, concepts may represent the meso-perspective, and communication may represent the micro-perspective. Regarding interrogation success, we propose the following:

*Proposition 2a. There is a positive (linear) relationship among the individual competencies that police officers rely on, the concepts of interrogation available to police officers, the number of applicable communication tactics and techniques, and interrogation success.*

*Proposition 2b. The level of competencies acquired determines the elaboration of interrogation concepts that, in turn, facilitate the application of communication tactics and techniques, which results in a successful interrogation.*

## Competence

The first pillar of police officers’ interrogation expertise is represented by their *competencies*. Briefly, competencies are defined as an active application of knowledge in applied settings (Leisen, 2010); that is, the police officers’ personal, professional, social, and methodical capabilities. In this context, competencies may comprise either general or specific knowledge, skills, abilities, and other characteristics, such as traits, interests, or attitudes (so-called KSAOs; Krumm et al., 2016). From an organizational psychological perspective, KSAOs as person-related capabilities may fit work-related job demands. In this vein, we conceptualize interrogations as specific work situations that offer specific job demands, which require specific KSAOs. Consequently, a good fit between person-related capabilities and job demands may promote success at work, including interrogation success (cf. Kristof-Brown et al., 2005; Kristof-Brown and Guay, 2011). Likewise, McClelland (1973) considers competencies as personal characteristics that help employees to successfully execute a task in a specific organizational context. Within the policing context, he proposes undertaking a job analysis to determine the most relevant job characteristics and competencies required.

If you want to test who will be a good policeman, go find out what a policeman does. Follow him around, make a list of his activities, and sample from that list in screening applicants. Some of the job sampling will have to be based on theory as well as practice. If policemen generally discriminate against blacks, that is clearly not part of the criterion because the law says that they must not. So include a test which shows the applicant does not discriminate. Also sample the vocabulary he must use to communicate with the people he serves since his is a position of interpersonal influence—and not the vocabulary that men who have never been on a police beat think it is proper to know. And do not rely on supervisor’s judgments of who are the better policemen because that is not, strictly speaking, job analysis but analysis of what people think involves better performance (McClelland, 1973, p. 7–8).

### Natural Competencies

In general, one may distinguish between three broad clusters of competencies: natural, explicit, and implicit competencies. First, person-related capabilities can be conceptualized as *natural competencies*, in terms of “innate” abilities. For instance, according to the theory of the “great man” (e.g., Hook, 1957), interrogation experts achieve interrogation success by applying their natural abilities. Generally, one would accept the existence of natural features, such as personality traits and intelligence, that are beneficial for interrogation success. For instance, cognitive capabilities, such as fluid intelligence, processing speed, and working memory, are important for effectively observing and managing the communication process (cf. Schneider and McGrew, 2012). In police service, high-performing police officers are characterized by high consciousness, high extraversion, and low neuroticism (Detrick and Chibnall, 2006, 2013). Additionally, individual differences in communication behavior may exist (e.g., Schulz von Thun, 1981b). However, the natural capabilities of humans may also be detrimental to interrogation success. For example, police officers’ impulsive and aggressive behaviors due to high neuroticism may put a strain on relationships with witnesses or suspects and interrupt the establishment of rapport (e.g., Ono et al., 2011).

Aside from natural competencies, such as personality and intelligence, interrogators are often susceptible to a wide range of “natural” biases and heuristics of human information processing that likely affect interrogation success. Cognitive biases, heuristic judgments, and decision-making are inherent mechanisms of human information processing and natural features of human cognition. Thus, the interrogation process is determined by cognitive biases and heuristics of both the interrogator and the respondent. For instance, confirmation bias refers to “the seeking or interpreting of evidence in ways that are partial to existing beliefs, expectations, or a hypothesis in hand” (Nickerson, 1998; p. 175). Human information processing involves both the seeking of information, which confirms interrogators’ beliefs, and the avoidance of information, which disconfirms an individual’s beliefs (Hill et al., 2008). Consequently, interrogators’ presumptions are crucial to the guilt of suspects. In fact, one might expect that interrogators’ information processing is originally biased by different sources (e.g., Lidén et al., 2018), and such biases may disturb and undermine interrogation success (e.g., Bond and DePaulo, 2008). Alongside confirmation bias, an interrogator’s accuracy of detecting the truth is usually higher than their accuracy of detecting lies (“truth bias”; Vrij and Baxter, 1999). In terms of demeanor bias, witnesses, victims, or suspects may appear more or less honest depending on their physical appearance or expressive style (DePaulo and Rosenthal, 1979). The phenomenon of illusory causation (Taylor and Fiske, 1975; McArthur, 1980) highlights the impact of an individual’s visual conspicuousness. If an individual’s visual conspicuousness increases, they are incorrectly perceived by observers as playing a causal role in an outcome, purely because of the saliency of their actions (Taylor and Fiske, 1978; McArthur, 1981). The emotional victim effect is described as the effect of a victim’s emotionality or demeanor on perceived credibility (Ask and Landström, 2010). In this context, the expectancy-violation theory (e.g., Burgoon and Hale, 1988) states that violations of social behavior norms and expectations affect the relationship between the violator and the opponent. Taken together, natural capabilities can either help or hinder an interrogator to achieve interrogation success. Consequently, we argue that natural competencies are limited to capabilities that promote favorable interrogation outcomes, such as high consciousness, high extraversion, and low neuroticism (cf. Detrick and Chibnall, 2006, 2013).

### Explicit Competencies

Individuals may acquire a wide range of *explicit competencies* based on their natural capabilities during their lifespan. According to White (1959), competence is considered the “effective interaction (of the individual) with the environment” (p. 317). The definition of competence according to Weinert (1999, 2001a,2001b,2001c) is widely used in applied contexts. Competencies are “learnable cognitive abilities and skills, which are needed for problem-solving as well as the associated motivational, volitional, and social capabilities and skills, which are essential for successful and responsible problem-solving in variable situations” (Weinert, 2001a, p. 27). This functional approach refers to the competencies that are assumed to be prerequisites for adequate functioning on the job and the learnable adaptability to job demands. In educational psychology and pedagogy, four sub-clusters of competencies have been established: professional competence, personal competence, social competence, and methodological competence (Roth, 1971; Klafki, 1972; Klieme, 2004). Such competencies may be accumulated either via vocational training and studies at the beginning of the professional career or advanced training and further education on and off the job.

In the policing context, *professional competencies* are necessary to accomplish the job demands of police services in a legally compliant manner (Thielgen, 2016). In the interrogation context, professional competencies comprise basic knowledge of the law and psychology. Knowledge of criminal law and legal criteria is needed throughout the interrogation process and includes adequate instruction concerning the time for and manner of appealing a decision and the differentiation between legal and prohibited interrogation methods (e.g., Habschick, 2016; Artkämper and Schilling, 2018; Niegisch and Thielgen, 2022; cf. Milne et al., 2003). Criminological knowledge refers to the causation of criminal acts, such as aggression and violence (e.g., Anderson and Bushman, 2002; Finkel and Hall, 2018), central risk factors of crime famously summarized as “Central 8” (e.g., Bonta and Andrews, 2016; Bartol and Bartol, 2021), recidivism (e.g., Andrews et al., 1990, 2006; Ward, 2002), and the development of delinquency (e.g., Farrington and West, 1990). Forensic knowledge refers to credibility criteria for the initial credibility assessment of suspects (Greuel et al., 1998; Ludewig et al., 2011). Psychological knowledge refers to human cognition, particularly the biased functioning of memory, attention, and decision-making (e.g., Shiffrin and Atkinson, 1969; Tversky and Kahneman, 1974; Simons and Chabris, 1999; Engle, 2002; Heubrock, 2010; Baddeley et al., 2014; Volbert and Banse, 2014; Volbert and Steller, 2014), and the formation of false memories (e.g. Loftus and Palmer, 1974; Ceci and Bruck, 1995; Garry et al., 1996; Mazzoni and Memon, 2003; Loftus, 2005; Shaw and Porter, 2015). Police officers must be particularly aware of the developmental effects of memory in childhood and the older population (e.g., Sporer and Martschuk, 2013), mental disorders (e.g., Lau et al., 2008; Volbert and Lau, 2013), and psychic trauma (e.g., Volbert, 2006), such as amnesia (e.g., Pyszora et al., 2003; Merckelbach et al., 2007). Moreover, regarding the credibility of witnesses’ testimony and suspects’ confessions, interrogators need to be sensitive to both suggestibility (e.g., Stern, 1904; Köhnken, 1997; Volbert, 1997; Gudjonsson, 2003, 2018; Kraus et al., 2016) and false confessions (e.g., Gudjonsson, 2003, 2018; Houston et al., 2014; Meissner et al., 2015; Volbert et al., 2019; Eidam et al., 2020; Gudjonsson et al., 2021).

*Social competence* refers to the ability to interact with others. For instance, Kanning (2002, 2007, 2009) differentiates “social orientation” (e.g., perspective-taking and active listening), “offensiveness” (e.g., willingness to conflict and assertiveness), “self-regulation” (e.g., self-control and adaptability), and “reflexibility” (e.g., social perception and covert influence). An interrogator’s mindset is thought to profoundly influence the nature of relationship management. In policing, the difference between warrior and guardian mindsets of police officers is important (Rahr and Rice, 2015; Stoughton, 2016). Police officers adopting the guardian mindset favor community-based policing and principles of procedural justice, which promotes the use of de-escalation communication techniques during police-citizen encounters. In contrast, police officers who rely on the warrior mindset adopt a more aggressive style of policing to establish authority as crime-fighting soldiers (McLean et al., 2019; Carlson, 2020; Clifton et al., 2021). Because of the immigration and refugee movements, the importance of intercultural skills has also been highlighted in police service. For instance, interrogation regarding cultural differences may affect the rapport phase (Heubrock, 2013). Moreover, the involvement of an additional interpreter may alter the dynamics of the interrogation (Schumann, 2019; Vrij and Leal, 2020).

*Personal competence* in police services refers to the individual characteristics that enable police officers to act independently, responsibly, and in a goal-oriented manner. In general, relatively stable characteristics, such as cognitive abilities, and certain personality traits are important prerequisites for police officers (Thielgen, 2016). In particular, core self-evaluations, vocational interests in police service, achievement and affiliation motivations, emotional stability, general mental abilities, and physical fitness are predictors of professional success (Nettelnstroth et al., 2019). Such general characteristics may also be evident in interrogations. Indeed, these findings correspond with the job profiles of police officers worldwide (e.g., in the USA; Aamodt, 2004; Davis and Rostow, 2014). Notably, personal competency may also be reflected in the appearance of police officers (i.e., their uniforms). For example, studies have shown that adequate personal appearance of police officers promotes perceptions of competency, respect, and trust, even among criminal offenders (Thielgen et al., 2020), and reduces perceptions of threat among civilians. Thus, it might be assumed that interrogators wearing business clothes would have similar effects.

Finally, *methodological competence* generally refers to the ability of knowledge acquisition. On a basic level, it comprises reading and writing skills. At an advanced level, this competency refers to analytical thinking and problem-solving skills. Reasoning and action strategies are aimed at the application of scientific procedures, such as observation, categorization, inductive and deductive reasoning, evaluation, and interpretation, in practical contexts. Indeed, methodological competencies are required for implementing professional (i.e., transferring theory to practice), social (i.e., interacting constructively), and personal capabilities (i.e., self-reflection). Police services and especially interrogations are usually conducted face-to-face, and thus the application of social, personal, and professional competencies to varied police operations and criminal cases is required. For instance, when interviewing violent offenders, police officers need to apply the psychological knowledge mentioned above during interrogations to recognize the accumulating risk factors for aggression (i.e., retention or imprisonment). In case of an attack, self-defense skills in dynamic environments must be applied appropriately (Staller and Körner, 2020). When interviewing children or victims of a crime, there is a special need for empathic care. However, police officers’ situational awareness may be reduced by the daily routine or long periods of inactivity (Rowe and Rowe, 2021). Finally, interrogators require different information technology skills, such as typing, audio and video recording, and preparation of interrogation protocols (Niegisch and Thielgen, 2022).

### Implicit Competencies

During the lifespan, learning processes occur both explicitly and implicitly (cf. Mazur, 2006). Criminological knowledge that is implicitly acquired via experiences during professional life is considered implicit competence. Specifically, implicit theories, reasoning, and intuition are understood as common elements of human decision-making and performance (e.g., Tversky and Kahneman, 1974; Gigerenzer, 2007; Kahneman, 2011; Harteis and Billett, 2013). According to Harteis and Billett (2013), “intuition is usually defined as the capability to act or decide appropriately without deliberately and consciously balancing alternatives, and without following a certain rule or routine, and, possibly, without awareness […]. It is commonly held to permit rapid reactions that result in effective outcomes” (p.146). Implicit information processing is particularly important when police officers need to recognize and evaluate certain abnormalities and coincidences (i.e., criminalistics serendipity; Holzhauer, 2016). Likewise, recent research on interrogation has become increasingly focused on implicit information processing. For instance, “pragmatic implication” describes the phenomenon whereby individuals process information “between the lines” and hear information as implied but not asserted (Redlich, 2019).

In summary, competencies help police officers to understand when and how to apply interrogation concepts (e.g., the extended cognitive interview) and communication techniques (e.g., specific questioning techniques) effectively (cf. Paulo et al., 2013). In regard to police officers’ interrogation expertise, native, explicit, and implicit competencies may induce additive and reciprocal effects that promote interrogation success (Dreyfus and Dreyfus, 1980; Ericsson et al., 2007; cf. Holzhauer, 2016). Thus, we propose:

*Proposition 3. Native, explicit (including methodical, professional, personal, and social competencies), and implicit competencies positively predict interrogation success.*

## Concept

The second pillar of police officers’ interrogation expertise is represented by concepts. In the interrogation context, concepts refer to planful and structured interview strategies. It is characterized by conscious and deliberative action plans, which guide the interrogation process of police officers. In general, the main goal of interrogation concepts is “to obtain information that is necessary for judicial criminal proceedings,” and “valid information as much as possible, for example, about the person making the statement, but also about other people, about the case, about courses of action, about locations” (Hermanutz et al., 2018; p. 11). However, interrogation concepts are typically designed for specific investigative context, i.e., for interviewing witnesses (Geiselman et al., 1985, 1986; Fisher et al., 1987, 2011; Fisher and Geiselman, 1992), victims (e.g., Bull, 2010; Fisher and Geiselman, 2010; Lamb et al., 2007), or suspects (Inbau et al., 2013). Regardless of the procedural status of civilians, we assume that an explicit, conceptually-based interrogation approach is always superior, in terms of interrogation outcomes, to a free interrogation approach. For example, the application of the (extended) cognitive interview for witnesses is associated with a significant increase in the number of correct details retrieved (Köhnken et al., 1999; Memon et al., 2010). Similarly, research from the USA indicates that both collaborative interrogation strategies (i.e., the information gathering-approach, such as the PEACE-model; Milne et al., 2003) and competitive interrogation strategies (the accusation approach, such as the REID method; Inbau et al., 2013) are associated with a higher likelihood of obtaining a confession (Meissner et al., 2014; Vrij et al., 2017b).

In standard interrogation situations, police officers may apply standard investigative concepts to criminal or administrative proceedings and crime prevention. Specifically, police officers need to be familiar with standard concepts, such as the (extended) cognitive interview for witnesses (Geiselman et al., 1985, 1986; Fisher et al., 1987, 2011; Fisher and Geiselman, 1992), the (extended) cognitive interview for suspects (Geiselman, 2012), structured interrogation for witnesses (Berresheim and Weber, 2003; Weber et al., 2011; cf. Weber and Berresheim, 2001) and suspects (Adler and Hermanutz, 2009; Berresheim and Capellmann, 2013), and the use of interviewing cards (Adler and Hermanutz, 2010, 2012, 2013).

Specific interrogation concepts have been designed to accomplish certain goals of the police service. In cases of major incidents, the independent interviewing protocol for eyewitnesses may help address the lack of information and gain investigative hypotheses (Krix and Sauerland, 2011; Hahn, 2017; cf. self-administered interview, Gabbert et al., 2009; Hope et al., 2011). When personnel resources are limited, a modified version of the cognitive interview offers an efficient survey strategy approach, particularly for ad recordings (e.g., modified cognitive interview for frontline police investigators; Dando et al., 2008). When police officers interview crime victims, specific interview concepts that fulfill the needs of vulnerable witnesses are crucial to minimize the adverse impact of secondary victimization (e.g., Bull, 2010; Fisher and Geiselman, 2010; Lamb et al., 2007). When police officers are required to interview individuals who speak a foreign language and have cultural barriers, specific effects related to the involvement of interpreters must be considered (Heubrock, 2013; Schumann, 2019; Vrij and Leal, 2020).

Beyond explicit interrogation concepts, concepts can comprise both explicit and implicit interrogation strategies. Police officers may use individual communication scripts as implicit concepts acquired during the course of their professional lifespan. Specifically, these concepts may be shaped by the accumulation of criminalistic experience within specific job domains, such as a criminal department involved in homicide investigations. Indeed, police officers’ individual and implicit communication strategies may significantly impact their behavior when interviewing witnesses, victims, and suspects. If implicit concepts are based on accumulated criminalistic experience, police officers are likely to achieve superior interrogation outcomes. For instance, such implicit concepts may be apparent in naturalistic decision-making (cf. Orasanu and Connolly, 1993; Zsambok and Klein, 1997; Klein et al., 2015) or criminalistic serendipity (e.g., Holzhauer, 2016). The importance of implicit empirical knowledge beyond explicit knowledge has been scientifically recognized (cf. Kahneman, 2011; e.g., Redlich, 2019), where implicit knowledge is understood as a special type of crystallized intelligence (Schneider and McGrew, 2012). Overall, the concepts of interrogation form a framework that guides police officers to use various levels and techniques of communication in a goal-oriented manner. Therefore, we propose:

*Proposition 4: Both explicit and implicit interrogation concepts predict interrogation success.*

## Communication

Communication represents the third pillar of police officers’ interrogation expertise and refers to the processes of sending and receiving information (i.e., information exchange) and mutual understanding (i.e., information congruency) (e.g., Shannon and Weaver, 1949; Schulz von Thun, 1981a; Watzlawick et al., 2011). Information science and psychology have offered basic theories and models of nonverbal and verbal communication that describe general interactions between people, particularly those during interrogations. For example, for nonverbal communication, the systems model of dyadic nonverbal interaction (Patterson, 1984, 1995, 2019) conceptualizes parallel sending and receiving of nonverbal processes between interacting individuals within a broad and dynamic ecological system. According to the model, communication takes place beyond the coordination of both partners’ contributions to the interaction. Specifically, the relationships between individuals’ characteristics and processes and the social ecology of the interaction setting are reciprocal and best analyzed at the systems level. Indeed, this approach is particularly relevant for interrogations to describe the dynamic interplay among individual, dyadic, and environmental processes during nonverbal interactions. This includes facial expressions (e.g., eye movements and facial expressions), voice, body movements, gesture and posture, proximity and distance, touch, and physical appearance (Knapp et al., 2014; Lorei and Litzke, 2014; Eaves and Leathers, 2018; Thielgen et al., 2020, 2022; Telser et al., 2022). Regarding verbal communication, different communication facets must be differentiated (e.g., content, relationship, appeal, self-disclosure, and the “four sides of a message” model; Schulz von Thun, 1981a; face work and the “S.A.V.E.” model; Rogan and Hammer, 2002). Moreover, it is important to observe communication from different levels (e.g., “interaction games” and the “model of dual-action regulation”; Sachse, 2013, 2019).

As an extension of the basic theories of communication, cognitive psychology offers a sound understanding of human information processing during interactions, including the pathways along which information is processed and the factors of distortion. For example, Keßler (1988) described a framework for psychological interviews, which suggested that the interviewing process leads to significant differences between cases experienced by civilians and cases documented by police officers. On the one hand, civilians’ mental processes of sensation, perception, memory, social cognition, cognitive bias, decision-making, and language may determine the information reported. On the other hand, the above mental processes of police officers may also determine the information documented. Thus, several sources of error can contribute to the differences between an experienced and a documented case (e.g., Steller and Volbert, 1997, for a review).

Finally, communication science and psychology offer a wide range of techniques that influence interaction processes. In the context of interrogations, techniques may focus either on information acquisition or relationship management (“individual methods of information gathering”; Kelly et al., 2013, and “rapport-building”; Kelly et al., 2013, 2015, 2016). Similar to craftsmen’s tools, communication and interrogation techniques fulfill specific purposes (e.g., Soukara et al., 2009; Cleary and Warner, 2016). Regarding witnesses, the concept of the (extended) cognitive interview encompasses the mental reinstatement of environmental and personal contexts (e.g., Fisher and Geiselman, 1992; Fisher et al., 2011). Regarding victims, scholars have developed techniques that aim to either reduce stress (cf. Deffenbacher et al., 2004) or minimize the impact of suggestion on memory performance (cf. Loftus, 2005). Regarding suspects, minimization techniques, such as appealing to the suspect’s self-interest or conscience, offering rationalizations, their link to confessions, and other interviewing outcomes, are research topics that have gathered recent interest (Kelly et al., 2019; Luke and Alceste, 2020). To date, research has identified 71 techniques for questioning suspects categorized into six groups (Kelly et al., 2013, 2015, 2016; cf. Cleary and Warner, 2016): rapport and relationship building (e.g., using similar language as the source), context manipulation (e.g., proximity and distance; c.f. Kelly et al., 2021), emotion provocation (e.g., reducing fear), collaboration (e.g., transfer of control over the interaction to the source), confrontation/competition (e.g., expression of anger), and presentation of evidence (e.g., concrete evidence).

Based on the goal of obtaining “as much undistorted information as possible” (Hermanutz et al., 2018), communication techniques can be classified into broad strands in the context of interrogation. Specifically, Meissner et al. (2021) proposed a model discussion of three fundamental challenges for eliciting the truth: (a) investigative biases, (b) the frailty of human memory, and (c) resistance to providing information. In this context, investigative interviewing encompasses both relational tactics (e.g., developing rapport and trust) and informational tactics (e.g., eliciting accurate information and facilitating judgments of credibility). From our point of view, we propose further differentiating the two strands of relational and informational interventions into four strands: the assessment and promotion of the ability for testimonies and the assessment and promotion of the motivation for testimonies.

### Ability for Testimony

We define the ability for testimony as the capability of the witness, victims, or suspects to provide as much unaltered information as possible. This includes adequate sensory perception, the ability to store, retrieve, and assign information in or from memory, the linguistic ability for verbal expression, the ability to control suggestive influences, and the mastery of relevant communicative skills (Volbert, 2005; cf. Loohs, 2013).

#### Assessment of the Ability for Testimony

During the interrogation process, police officers should *assess the ability for testimony* of their interaction partners, which may be documented as a behavioral observation in an impression note (Niegisch and Thielgen, 2022). A prerequisite for a sound assessment is basic knowledge of several occasions in which the informative value may be impaired (Daber, 2003). Various factors can impair the ability for testimony: the level of development (e.g., children and adolescents), cognitive abilities (e.g., intellectual disabilities), abnormalities in experience and behavior (e.g., perception, storage, and recapitulation of experienced episodes), indications of psychopathological issues (e.g., organic brain impairments, neurological disorders, drug or alcohol abuse, acute intoxication at the time of the crime, or delusions and dissociations), and personality disorders (cf. Daber, 2003; cf. Hermanutz et al., 2018). Moreover, police officers should be aware of the mutual influence of co-witnesses or the influence of the media after an event (Paterson and Kemp, 2006).

Police officers may use several techniques to assess the ability for testimony. For instance, basic knowledge of behavioral observation helps police officers establish a sound baseline (Hermanutz and Adler, 2011; Schemmel and Volbert, 2017; c.f. Faßnacht, 1995). Moreover, the detection of psychopathological symptoms is important for impression notes, such as delusions and hallucinations (Niegisch and Thielgen, 2022; cf. Arbeitsgemeinschaft für Methodik und Dokumentation in der Psychiatrie [AMDP], 2018).

#### Promotion of the Ability for Testimony

The *promotion of the ability for testimony* may be at the core of interrogation success. For this purpose, police officers may use effective communication techniques in a goal-directed manner. Both a high number of correctly reported details and a low number of incorrectly reported details or errors characterize good memory retrieval performance (i.e., false, altered, or invented information). In this context, it would be effective to apply both general communication techniques (e.g., active listening skills; Kimai and Groß, 2014) and specific techniques that promote memory retrieval performance of the witnesses, victims, or suspects (cf. Krix and Sauerland, 2013).

Police officers may use several basic empirically supported techniques to promote the retrieval process. Initially, the interrogator may use techniques of the (extended) cognitive interview (e.g., Fisher and Geiselman, 1992; Fisher et al., 2011). Scholars consider the application of the free report (in-depth reporting) as a gold standard (e.g., Berresheim and Weber, 2003; Hermanutz and Litzcke, 2012; Meissner et al., 2015). Subsequently, interrogators may continue using a funnel-shaped question strategy, whereby open-ended questions are asked at the beginning, and closed-ended questions are asked at the end of the interview. However, police officers should avoid suggestive questions (Loftus, 2005; Arntzen, 2009; Heubrock and Donzelmann, 2009; Reinhold et al., 2016). Moreover, they should be cautious when asking questions about hypothetical evidence to prevent inflation of perceptions of guilt and memory distortions (Crozier et al., 2020).

As an extension of basic query strategies, the use of retrieval aids is effective. For example, the mental reinstatement of environmental and personal contexts or describing the event in several orders (e.g., Fisher and Geiselman, 1992; Fisher et al., 2011; cf. Köhnken et al., 1999; Memon et al., 2010). In terms of content, different systems of perception, so-called representation systems, should also be addressed, such as auditory, tactile, and/or olfactory information (Bandler and Grinder, 1981; Ötsch and Stahl, 1994; cf. Fisher and Geiselman, 1992; Fisher et al., 2011).

Recently, innovative techniques have been added to the best practices of interrogations (e.g., Artkämper, 2009a,b). For example, a “familiar interrogation atmosphere” (Reutermann, 2015, S. 103) may reduce stress and significantly increase memory retrieval performance (Deffenbacher et al., 2004). An empirically proven method for maximizing the amount of correct visual and auditory information obtained when dealing with memory gaps is eye closing, which can be effectively combined with focused mediation (Perfect et al., 2008; Wagstaff et al., 2011; Vredeveldt et al., 2015). Moreover, research recommends that memory content should be structured. For instance, police officers may ask witnesses to present their statement in chronological order during a second free-report phase using the timeline technique (Kahana et al., 2008; Unsworth, 2008; Hope et al., 2013). Finally, the category clustering technique may help organize content according to people, objects, location, action, conversations, and sounds (Paulo et al., 2016, 2017).

### Motivation of Testimony

We define motivation for testimony as the willingness of the witness, victim, or suspect to provide as much information as possible. In the case of a suspect, motivation for testimony may be associated with a confession (cf. Gudjonsson, 2003, 2018, 2021, for a review). Various factors may be associated with high motivation for testimony. For witnesses and victims, important aspects include the need for justice, punishment of the perpetrator, and securing claims under civil law. In contrast, important aspects for suspects may include aversion or mitigation of punishment or “wanting to explain oneself.” However, while interacting with others, people also strive to fulfill so-called relationship motives, such as affiliations or power (cf. McClelland, 1987). These motives can be broken down into six “interactional goals”: “recognition,” “importance,” “reliability,” “solidarity,” “autonomy,” and “territoriality” (Sachse, 2013, 2019). Indeed, interactions between police officers and civilians are typically complimentary because of a hierarchical gradient (police authority versus interviewed person). However, in some cases, a symmetrical or even submissive communication style by police officers may fulfill the motives or interactional goals of the communication partner. Consequently, the rapport between interaction partners may enhance the willingness to disclose information and thus promote interrogation success (cf. Sachse, 2013; Sachse, 2019).

#### Assessment of the Motivation for Testimony

In the course of the interrogation process, police officers should *assess* witnesses’, victims’, or suspects’ *motivation for testimony*. This is particularly important for differentiating truth or error from lies (Hermanutz and Litzcke, 2012). Although errors are typically associated with the ability for testimony, there is a close link between deception and the motivation for testimony. Indeed, lies can help interviewees gain advantages on a personal or procedural level. In some individuals, false statements are even more likely to be made. For instance, people with narcissistic, histrionic, or borderline personality disorders may provide false statements to fulfill implicit relational motives (e.g., confabulations; cf. Sachse, 2013). Moreover, people with antisocial personality disorder may report false statements as part of the disorder (cf., Böhm et al., 2002).

The assessment of the motivation for testimony is demanding and often inaccurately assessed in practice (Aamodt and Custer, 2006). For example, practitioners frequently hold misbeliefs about the relationship between nonverbal behaviors and deception (i.e., the diagnostic value of nonverbal cues to detect deception; Vrij et al., 2019). However, nonverbal cues to deceit are subtle and unreliable (DePaulo et al., 2003), and several problems are associated with the assessment of nonverbal behaviors (e.g., cues and clusters that may be easily overlooked, unprecise measurement of cues, idiosyncratic behaviors, contagious behaviors, and situational differences; Vrij et al., 2019). Instead, the interrogator should focus on the verbal content of the testimony (DePaulo et al., 2003), particularly via the application of credibility criteria (Daber, 2015; Hauch et al., 2016). This approach is based on the idea that a higher number of indicators makes a statement more credible (Greuel et al., 1998). For instance, truth-tellers provide more verifiable details than liars, which supports the so-called verifiability approach (e.g., Vernham et al., 2020; Palena et al., 2021b).

To assess the motivation for testimony, police officers can use several basic techniques. For instance, the baseline technique offers the possibility of a cased-independent assessment of the interviewee’s communication style (Hermanutz and Adler, 2011; Schemmel and Volbert, 2017). Notably, a baseline should be based on not only small-talk but also the exploration of peoples’ personal backgrounds (cf. Vrij et al., 2019). Moreover, a thorough free (in-depth) report may provide a sound basis for subsequent evaluation of motivation of testimony via a credibility assessment. In such cases, audio-visual files allow police officers to review the interrogation afterward. For repeated interrogations, assessing the consistency of statements can provide additional insights (Offe and Offe, 2008). Furthermore, in repeated interviews, free reports may be particularly effective if the interrogator provides an immediate “report everything” instruction, which can help police officers discriminate between truthful and deceptive accounts more effectively (Izotovas et al., 2020).

More elaborate approaches that extend on basic techniques aim to improve the accuracy of discriminating truths from lies (Breuer and Cramer, 2011; Sporer, 2016). Specifically, these techniques are based on the basic principle of provoking cues for true or false statements to amplify the differences between liars and truth-tellers (Vrij and Granhag, 2012, 2014). Techniques that require a high cognitive load have been shown to be effective (e.g., Vrij et al., 2008a,b). To increase cognitive load, police officers can use specific types of questions, such as asking the interviewee to report the case in reverse order (Vrij et al., 2008a,b, 2012; cf. Vrij et al., 2017a) or asking unexpected questions (cf. Vrij, 2015, for a meta-analysis). Additionally, police officers can encourage the interviewee to provide even more comprehensive statements to generate a broader information base. This is also the case for the use of unexpected targeted questions (Vrij et al., 2009; Vrij and Granhag, 2014; cf. Vrij et al., 2017a). Finally, the strategic use of evidence may also facilitate lie detection (e.g., Hartwig et al., 2006; cf. Vrij et al., 2017a) because the interviewee’s reaction toward evidence may reveal additional cues for truths or lies.

Recent research has proposed more innovative and creative techniques, such as “model statements” (Leal et al., 2015). To foster cues of deception, police officers may prepare civilians for interrogation by presenting videotapes of a truthful and detailed account of an event unrelated to the case (cf. Leal et al., 2021). In addition, classical approaches, such as free recall, may be combined with innovative techniques, such as model statements (Leal et al., 2015) or sketching (Vrij et al., 2020), to promote information elicitation and lie detection over multiple interviews (Deeb et al., 2021).

#### Promotion of the Motivation of Testimony

Another central task of a successful interrogator is to *promote the motivation for testimony* through the conscious use of communication. Similar to the promotion of the ability of testimony, promotion of the motivation for testimony is at the core of interrogation success, and there are several effective basic communication techniques (Rogers, 1983; Kimai and Groß, 2014). As a key element of interviews (St.-Yves, 2006), the establishment of rapport is generally accepted as “a celebrated aspect of modern interrogation practice” (Meissner et al., 2014, S. 23). Rapport can be promoted by both nonverbal (Collins et al., 2002) and verbal approaches (Krix and Sauerland, 2013; Vallano and Schreiber Compo, 2011). Moreover, relationship-based or procedurally-based rapport can be established (Huang and Teoh, 2019). In particular, beneficial techniques are direct reactions to the other person, active listening, mirroring, self-revelation, showing similarities, continuous and repeated contact, and contrasting (Kelly et al., 2013; cf. Tickle-Degnen and Rosenthal, 1990). Above all, rapport is effective because it not only promotes sympathy and reduces stress (e.g., Collins et al., 2002; Fisher, 2010) but also offers basic relationship motives (cf. McClelland, 1987; Sachse, 2013, 2019).

In some cases, interviewees may be resistant, which may result in an initial failure to establish rapport. Here, resistance can be resolved by intensively responding to the other individual (Berresheim and Capellmann, 2013). A promising approach is the application of motivational interviewing, which has been successfully used in the context of the penal system (motivational interviewing; Miller and Rollnick, 2013; cf. Breuer et al., 2016). It is worth noting that in some cases, the interrogator may also be reluctant to establish rapport. For instance, the interrogator’s emotions may influence the goals and tactics of interrogations of suspects involved in child sexual abuse (Magnusson et al., 2021). Consequently, a change of the interrogator may be indicated.

As an extension of the basic techniques, the strategic use of evidence can also promote the motivation of interviewees to make statements (Hartwig et al., 2006, 2014; Granhag and Hartwig, 2015; cf. Gudjonsson, 2003). For instance, the presentation of evidence can indicate that the police have already acquired comprehensive information; consequently, withholding information or even lying would discredit the interviewee. In this context, recent research supports both late (Hartwig et al., 2014) and gradual presentation of evidence (cf. Meissner et al., 2014).

Taken together, we consider that the assessment and promotion of both the ability for testimonies and the motivation for testimonies may promote interaction success. Thus, we propose:

*Proposition 5. The assessment and promotion of the ability for testimony and the motivation for testimony predict interrogation success.*

## Organizational Context: Research, Training, Practice, and Selection

Typically, police officers acquire interrogation expertise through self-learning processes, observation of other police officers (Bandura, 1986, 2008), and accumulation of individual experiences of interacting with witnesses, victims, and suspects. Specifically, we propose four factors that promote police officers’ interrogation expertise: personnel selection, empirical knowledge and research findings, police officers’ vocational training and studies, and police practice and work experience. First, at the beginning of an occupational career, police officers and criminal detectives are usually required to undergo an in-depth *personnel selection procedure*. Based on the principles of the person-environment-fit approach (Kristof-Brown et al., 2005; Kristof-Brown and Guay, 2011; van Vianen, 2018), organizations need to select personnel to maximize the fit between the employee’s competencies and the characteristics of the job demands. Thus, police organizations should recruit candidates with sufficient potential to successfully complete vocational studies and training and sufficient capabilities to master occupational career demands (Thielgen, 2016; Schade and Thielgen, 2022).

Second, both practitioners and investigators seek effective methods and techniques to encourage witness testimonies and motivate suspects to confess. However, there is an ongoing debate among researchers concerning the optimal empirically-based interrogation strategies, tactics, and techniques in both Europe (e.g., Holzhauer, 2016; Sticher, 2007) and internationally (Laney and Loftus, 2016; Snook et al., 2021). *Empirical knowledge and research findings* have identified several factors that predict interrogation outcomes. For instance, an important example of the sophisticated approach is the extended cognitive interview (Geiselman et al., 1985, 1986; Fisher et al., 1987, 2011; Fisher and Geiselman, 1992).

Third, *police officers’ vocational training and studies* may help establish a minimum standard of interrogation skills. Additionally, advanced training and further education in line with job experience may help individuals achieve expert status. The typical learning processes of personnel development include formal instruction, scenario-based learning, learning by observation, training on the job, supervision, and reflection (Nerdinger et al., 2019). In the context of interrogations, interrogation training is an effective way to promote police officers’ interrogation success (e.g., Weber and Berresheim, 2001; Adler and Hermanutz, 2008; Sticher, 2017; Chevalier and Rüffer, 2020; Chevalier et al., 2020; Reinald et al., 2021; Körber et al., 2022).

Fourth, during their careers, police officers accumulate *practical work experience*, such as experience in interrogations. However, research suggests that the relationship between age indicators (e.g., work experience) and job performance is close to zero (Ng and Feldman, 2008). Both implicit and explicit learning processes may eventually lead to maladaptive and counterproductive behaviors, such as negligence in the informing of rights or unconscientious drafting of the interrogation protocol. According to the step model of expertise development by Dreyfus and Dreyfus (1980, 1991), individuals progress through a skill acquisition process, starting from the status of a beginner (novice) to the status of an advanced, competent, or experienced learner, and finally to an expert. Achieving expert status requires deliberate practice; that is, learning processes that are highly structured and geared towards continuous improvement in performance. Some scholars estimate that to achieve expert status, at least 10,000 h or 10 years of reflective practice in a specific job domain is needed (Ericsson et al., 2007).

Therefore, personnel selection may help police officer candidates acquire sufficient potential to develop interrogation expertise. Furthermore, empirical evidence may inform vocational studies and training toward better education and training concept designs for the police service. Finally, vocational studies and training, as well as occupational practice, provide police officers with expertise in specific job domains. Thus, policing studies, training, and practical experience may promote the development of interrogation expertise. In summary, we propose:

*Proposition 6. Four main contextual factors promote police officers’ interrogation expertise: personnel selection, empirical knowledge and research findings, vocational training and studies, and practical work experience.*

## Discussion

The primary topic of the present paper was interrogation as a core task of police work. Indeed, interrogations are an indispensable element of criminal investigations that sometimes provide the only evidence toward solving a case. The overarching aim of the present paper was to examine which factors contribute to successful interrogation. We argued that interrogation success does not depend on chance; rather, it is a result of the interplay among three elements of police officer interrogation expertise: competence, concept, and communication. We conceptualized these elements as inextricably linked, which corresponds to the current understanding of international interviewing research (Kelly et al., 2013, 2015, 2016; Meissner et al., 2021). A pure focus on competencies would be detached from the specific interviewing situation. Likewise, the sole application of communication techniques and models, without the consideration of competencies or conceptual control, would be arbitrary and could be detrimental to the process of investigation.

First, we introduced the term interrogation success, which was described by in-role and extra-role performance, the absence of counterproductive behaviors, and civilians’ favorable attitudes towards interrogation strategies (Proposition 1a), which fulfilled the criteria of objectivity, reliability, validity, and efficiency (Proposition 1b). Moreover, we expected a positive (linear) relationship between police officers’ interrogation expertise and interrogation success [i.e., individual competencies that police officers rely on, the concepts of interrogation available to police officers, and the range of communication tactics and techniques applicable (Proposition 2a)]. Thus, the level of competencies enables police officers to acquire elaborate interrogation concepts, which in turn, facilitate the application of communication tactics and techniques (Proposition 2b). Competencies include natural competencies and explicit (e.g., professional, methodical, personal, and social competencies) and implicit capabilities (Proposition 3). Concepts may be either explicitly or implicitly represented (Proposition 4). Furthermore, communication may focus on the assessment and promotion of the ability for testimony as well as the assessment and promotion of the motivation for testimony (Proposition 5). Finally, four main factors may promote police officers’ interrogation expertise: personnel selection, empirical knowledge and research findings, vocational studies and training, and police practice and work experience (Proposition 6). Taken together, the present paper informs practical police service and police training by offering an evidence-based interrogation standard.

### Limitations and Directions for Future Research

In the present paper, we primarily focused on the role of police officers’ interrogation expertise and its relationship with interrogation success. Specifically, we refer to the decision-making process of identifying and applying communication tactics and techniques based on individual interrogation concepts and competencies to meet the demands of the complex and dynamic interrogation context (i.e., a “police officer-centered” approach). However, interrogations in practice are complex and dynamic situations that take place in a specific environment. Indeed, recent systems models of dyadic communication (e.g., Patterson, 1984, 1995, 2019) have proposed that interviewees send and receive information simultaneously within a broader and more dynamic ecological system. Consequently, we should consider the individual characteristics and psychological processes of witnesses, victims, and suspects (i.e., a “citizen-centered” approach) and the social ecology of the interaction setting (e.g., a “systemic” approach) (e.g., Palena et al., 2021a). Moreover, we suggest that civilians (i.e., witnesses, victims, and suspects) also behave in accordance with their competencies, concepts, and communication tactics and techniques within the interrogation context. Indeed, recent research has become increasingly focused on civilians’ characteristics and their relationship with the interrogation success of police officers (cf. Figure 1). First, civilians’ competencies may have an impact on interrogation outcomes. For instance, a study by Vrij et al. (2010) pursued the question of what constitutes a good liar and suggested 18 attributes that may help people to be perceived as trustworthy. Important concepts include deception theory (Zuckerman et al., 1981), naive views on how liars respond (Vrij et al., 2006; Granhag and Hartwig, 2008; Vrij et al., 2008a,b), impression formation theory (Ambady et al., 2000), persuasion theory (DePaulo and Friedman, 1998), and other characteristics and strategies of good liars (e.g., personality, states such as cognitive load and emotions, and behaviors).

Second and third, civilians’ concepts and communication tactics and techniques, respectively, may have an impact on interrogation outcomes. Recent research has become increasingly focused on suspects’ counter-interrogation behaviors. This concept may be defined as tactics applied by suspects to withstand an interviewing situation and appear convincing (Clemens, 2013). Important strategies include “being honest,” “mentioning a lack of motive,” “making a truthful impression,” “anybody could have done it,” “distancing oneself from the criminal,” “being deceptive,” and other strategies. Indeed, a sound understanding of counter-interrogation behaviors is critical for adapting to effective interrogation strategies (Clemens and Grolig, 2019). Several studies have discussed ways to cope with such counter-interrogation strategies (e.g., the Scharff technique; Rantamäki et al., 2020). For instance, the Shift-of-Strategy (SoS) is an extension of the strategic use of evidence to cope with counter-interrogation strategies. Here, interviewers influence suspects’ strategies to encourage them to become more forthcoming with information by highlighting discrepancies between their statements and the available evidence, particularly in a non-accusatory manner (Luke and Granhag, 2020).

Fourth, contextual factors may also significantly impact interrogation outcomes. Recent research has increasingly focused on the effects of police officers’ interrogation context manipulations and their effect on interrogation outcomes (e.g., Kelly et al., 2021). However, civilians may also determine the interrogation context. For example, they may invite trusted people to their interrogation, such as family, friends, or lawyers, which alters the interrogation context.

Taken together, future research should conceptualize interrogations in a balanced manner, considering both the behavior and experience of police officers and civilians within a dynamic ecological system. We highlight that interrogations are highly demanding situations that require a meta-perspective to conduct effective “conversation management” (Shepherd and Kite, 1988). Police officers must pursue interrogation goals, observe the ongoing situation and interaction dynamics, and, if necessary, make adjustments to both the concept applied and the communication tactics and techniques used. Specifically, policed officers must monitor and adapt their own behaviors and experiences, especially with regard to their own actions, emotions, and motivations (cf. Frese and Zapf, 1994; Gross, 1998; Muraven and Baumeister, 2000). Indeed, this may be reflected in the typical dynamics of an interview: “interrogation techniques and counter-interrogation strategies can … be seen as an ongoing game of cat and mouse” (Granhag et al., 2015, S. 309).

## Conclusion

We emphasize that there is not one optimal interrogation method or technique. Rather, we describe the achievement of interrogation success as a course of personal and professional development. From our point of view, the organizational goal must be to systematically promote the development of three levels of competence, concepts, and communication: at the beginning of the police career by personnel selection, then by targeted teaching and training as part of policing studies and specific advanced training (e.g., Weber and Berresheim, 2001; Adler and Hermanutz, 2008; Sticher, 2017; Chevalier and Rüffer, 2020; Chevalier et al., 2020; Reinald et al., 2021; Körber et al., 2022) and finally by gaining several years of experience within a specific area of crime or phenomena or specific interviewing topics, such as lie detection (e.g., Vrij and Fisher, 2020). However, the most important consideration for the concept of police officers’ interrogation expertise is not to focus on the development from beginner to expert but rather, to stimulate discussion regarding a sound standard at the end of policing studies and the first years of service. Ultimately, this should take into account the expectations of the members of a professionally operating citizens’ police force.

## Author Contributions

MT and PN conducted a first literature review as basis of a manuscript by Niegisch and Thielgen (2018) in German language. SS extended the current literature review. MT and SS equally conceptualized the current theorizing and wrote the manuscript and equally contributed to editing the first draft to its final version within the peer-review process. All authors contributed to the article and approved the submitted version.

## Conflict of Interest

The authors declare that the research was conducted in the absence of any commercial or financial relationships that could be construed as a potential conflict of interest.

## Publisher’s Note

All claims expressed in this article are solely those of the authors and do not necessarily represent those of their affiliated organizations, or those of the publisher, the editors and the reviewers. Any product that may be evaluated in this article, or claim that may be made by its manufacturer, is not guaranteed or endorsed by the publisher.
